# Long-term fat redistribution in ARV-naïve HIV+ patients initiating a non-thymidine containing regimen in clinical practice

**DOI:** 10.7448/IAS.17.4.19553

**Published:** 2014-11-02

**Authors:** Elena Ferrer, Antonio Navarro, Jordi Curto, Pilar Medina, Nerea Rozas, Gladys Barrera, Maria Saumoy, Juan Manuel Tiraboschi, Carmen Gomez, Daniel Podzamczer

**Affiliations:** 1 Infectious diseases, Hospital Universitari de Bellvitge, Barcelona, Spain; 2 Rheumatology Department, Hospital Universitari de Bellvitge, Barcelona, Spain

## Abstract

**Introduction:**

Lipodystrophy is still a matter of concern in HIV+ patients receiving ART. However, long-term fat change in patients taking non-thymidine regimens is not well known.

**Materials and Methods:**

A prospective ongoing fat change assessment including clinical evaluation and dexa scans (Hologic QDR 4500) is being conducted in all consecutive patients initiating ART from January 2008. Arm, leg, trunk and total fat as well as fat mass ratio (FMR=% trunk fat/% leg fat) were determined. Patients with data at baseline (BL), 12 and 36 m are included in this analysis. ITT and OT were performed. Multivariate general linear models were used to assess changes in fat measures.

**Results:**

One hundred patients were included. 81% men, 42.9 years, 18% AIDS, CD4 218.5 (6-756), viral load 5 log (2.9-6.8), leg fat 4644g, trunk fat 6693g, FMR 0.94. Around 40 patients (40%) initiated a PIr (17 LPVr, 11 ATVr, 9 DRVr, 3 FPVr), 34 (34%) NVP and 21 (21%) EFV. About 83% received TDF/FTC and 10% ABC/3TC. Groups were comparable at BL except for a lower viral load in NVP patients (*p=*0.047) and lower c-LDL in PI patients (*p*=0.043). After 36 m, no patient presented a clinically evident lipodystrophy. At 12 m, an overall significant increase was found from baseline in trunk, leg and FMR (median 759 g, 479.4 g and 0.03, respectively, *p*<0.05) and at 36 m in trunk and leg fat (median 989.9 g, 566 g, respectively, *p<*0.05). According to ART, at 12 m a significant increase in trunk and leg fat was observed in EFV and PIr. At 36 m, in NVP patients trunk and leg fat as well as FMR increased, whereas in PIr patients only leg fat increased (see figure). In ITT analysis, adjusted by age, sex, risk practice and BL CD4, EFV was associated with a greater increase in FMR (*p*=0.036) at 36 m vs PIr. In OT analysis, at 12 m, NVP was associated with a smaller percentage increase in trunk fat (vs PIr and EFV, *p*=0.006) and in leg fat (vs PIr, *p*=0.046). These differences did not persist at 36 m.

**Conclusions:**

In this cohort of patients taking non-thymidine-based regimens, after 36 m without a clinically evident lipodystrophy, no significant changes in FMR were observed. However, some differences in fat redistribution according to ART were present: PIr was associated with an initial and continuous increase in trunk and leg fat, NVP with a slower and progressive increase in both fat compartments, while in EFV patients, the initial fat increase was followed by a decrease in peripheral fat at 36 m. Longer follow up will help to confirm these trends.

**Figure 1:**
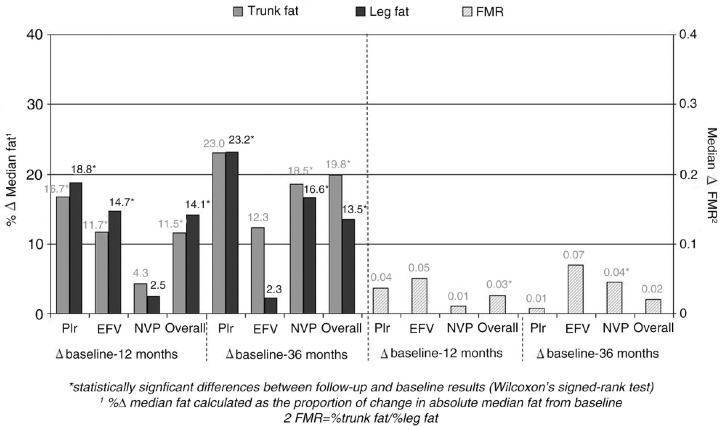
Changes in Trunk fat, Leg fat and FMR at 12 and 36 months, according to ART.

